# The global convergence of spectral RMIL conjugate gradient method for unconstrained optimization with applications to robotic model and image recovery

**DOI:** 10.1371/journal.pone.0281250

**Published:** 2023-03-16

**Authors:** Nasiru Salihu, Poom Kumam, Aliyu Muhammed Awwal, Ibrahim Mohammed Sulaiman, Thidaporn Seangwattana

**Affiliations:** 1 Center of Excellence in Theoretical and Computational Science, Fixed Point Research Laboratory, Fixed Point Theory and Applications Research Group, Faculty of Science, King Mongkut’s University of Technology Thonburi, Bangkok, Thailand; 2 Department of Mathematics, Faculty of Sciences, Modibbo Adama University, Yola, Nigeria; 3 Gombe State University Mathematics for Innovative Research Group, Gombe State University, Gombe, Nigeria; 4 KMUTT-Fixed Point Research Laboratory, Science Laboratory Building, Department of Mathematics, Faculty of Science, King Mongkut’s University of Technology Thonburi, Bangkok, Thailand; 5 Department of Medical Research, China Medical University Hospital, China Medical University, Taichung, Taiwan; 6 Department of Mathematics, Faculty of Science, Gombe State University, Gombe, Nigeria; 7 School of Quantitative Sciences, Universiti Utara Malaysia, UUM Sintok, Kedah, Malaysia; 8 Institute of Strategic Industrial Decision Modelling, Universiti Utara Malaysia, Sintok, Kedah, Malaysia; 9 Faculty of Science Energy and Environment, King Mongkut’s University of Technology, North Bangkok, Rayong Campus, Rayong, Thailand; Hebei University of Technology, CHINA

## Abstract

In 2012, Rivaie et al. introduced RMIL conjugate gradient (CG) method which is globally convergent under the exact line search. Later, Dai (2016) pointed out abnormality in the convergence result and thus, imposed certain restricted RMIL CG parameter as a remedy. In this paper, we suggest an efficient RMIL spectral CG method. The remarkable feature of this method is that, the convergence result is free from additional condition usually imposed on RMIL. Subsequently, the search direction is sufficiently descent independent of any line search technique. Thus, numerical experiments on some set of benchmark problems indicate that the method is promising and efficient. Furthermore, the efficiency of the proposed method is demonstrated on applications arising from arm robotic model and image restoration problems.

## Introduction

In unconstrained optimization, large scale problem of the following form

$$\begin{array}{c} \text{min}\;f\left(x\right),\;x\in R^{n}, \end{array}$$ (1)

is usually considered, where the function $f:R^{n}\to R$ is smooth, meaning its gradient, *g*(*x*) = ∇*f*(*x*) is obtainable. Problems in the form of Eq (1) attracted much attention in optimization community because of their wide variety of applications in different fields [1], including portfolio choice [2, 3], *m*-tensor system [4], image restoration [5–8], signal recovery [9–11] and robotic motion [12–14] among others. The iterative methods that make use of gradient of *f* are usually preferable to solve these problems. Example of such methods is the Conjugate Gradient (CG) method, owing to its modest memory requirement and nice theoretical property. The method generates sequence {*x*_*k*_} by taking $x_{0}\in R^{n}$ as an initial guess for the solution using the scheme

$$\begin{array}{c} \begin{array}{c} x_{k+1}=x_{k}+\alpha_{k}d_{k},\;\;k=0,1,2,\ldots . \end{array} \end{array}$$ (2)

The scalar *α*_*k*_ > 0, called step-size or line-step is calculated so that it approximately meets the condition

$$\begin{array}{c} f\left(x_{k}+\alpha_{k}d_{k}\right)\approx \underset{\alpha \geq 0}{\text{min}}\{f(x_{k}+\alpha d_{k})\}. \end{array}$$ (3)

A practical technique used for obtaining the step-size is Wolfe rule [15]. The technique at *k*-th iteration is computed in such away that *α*_*k*_ satisfies certain defined rules [16]. The commonly used technique referred as the standard line search technique, consisting the following inequalities

$$f(x_{k}+\alpha_{k}d_{k})\leq f\left(x_{k}\right)+\delta \alpha_{k}g_{k}^{T}d_{k},$$ (4)

$$g{\left(x_{k}+\alpha_{k}d_{k}\right)}^{T}d_{k}\geq \sigma g_{k}^{T}d_{k}.$$ (5)

Replacing Eq (5) with the formula

$$\begin{array}{c} |g{\left(x_{k}+\alpha_{k}d_{k}\right)}^{T}d_{k}|\leq -\sigma g_{k}^{T}d_{k}, \end{array}$$ (6)

gives the rule called strong Wolfe condition, where 0 < *δ* < *σ* < 1. Moreover, this step-size is calculated along the spectral search direction *d*_*k*_, given by

$$\begin{array}{c} d_{k}=\{\begin{array}{cc} -g_{k}, & if\;\;k=0,\\ -\theta_{k}g_{k}+\beta_{k}d_{k-1}, & if\;\;k\geq 1, \end{array} \end{array}$$ (7)

in which, the spectral and CG updating parameters are represented by the scalars *θ*_*k*_ and *β*_*k*_ respectively. The parameter *β*_*k*_ is crucial in constructing and determining choice of a CG method. Some earlier formulas for the parameters are proposed by Fletcher and Revees (FR) [17] and Polak, Ribière and Polyak (PRP) [18, 19] with the following formulas respectively

$$\begin{array}{c} \beta_{k}^{FR}=\frac{\|g_{k}{\|}^{2}}{\|g_{k-1}{\|}^{2}},\;\;\;\;\beta_{k}^{PRP}=\frac{g_{k}^{T}y_{k-1}}{\|g_{k-1}{\|}^{2}}, \end{array}$$

with *y*_*k*−1_ = *g*_*k*−1_ − *g*_*k*_ and ‖.‖ represents *ℓ*_2_ norm.

Others include; Hestenes and Steifel (HS) [20], Liu and Storey (LS) [21], Dai and Yuan (DY) [22], and Conjugate Descent (CD) [23]. It is observed in theory that, if the underlying quadratic function happen to be strongly convex, and *α*_*k*_ is determined by Eq (3), then the aforementioned schemes are comparable. However, for non-quadratic functions that mostly come up in reality, the behavior of the methods differs. For instance, when the difference between two consecutive gradients tends to zero, then, the PRP, HS and LS methods automatically performs a restart. This feature makes them numerically effective. However, they are theoretically unstable, and require some variants of Wolfe-type line search rule to converge. On the other hand, the FR, DY and CD methods have stable convergence property with modest numerical results [24].

The convergence result of FR scheme via the exact line search was initially discussed by Zoutendijk [25]; where Al-Baali [26], Touati-Ahmed and Storey [21], and Gilbert and Nocedal [27] further analyzed the theoretical result under the strong Wolfe conditions. Similar result was also recorded for the CD and DY methods. Furthermore, survey of literature revealed that the PRP method is one of the most reliable CG methods. However, Powell [28] showed that the method possesses unstable global convergence property. This led to several modifications of the method by different authors. For example see, [27, 29–32] among others.

Consider the modified version of the PRP method proposed by Rivaie et al. [33]

$$\begin{array}{c} \beta_{k}^{RMIL}=\frac{g_{k}^{T}y_{k-1}}{\|d_{k-1}{\|}^{2}}. \end{array}$$ (8)

The RMIL method has been given considerable attention recently, but unfortunately, the convergence result of the RMIL is established based on exact line search, which is sometimes difficult to obtain. It has been pointed out by Dai [34] that the RMIL method may not generate descent directions for general functions, i.e.

$$\begin{array}{c} g_{k}^{T}d_{k}\leq -\eta {\left\|g_{k}\right\|}^{2},\;\;\eta >0, \end{array}$$ (9)

may fail to hold. Subsequently, based on the condition imposed on RMIL method by Dai [34], i.e. $0\;\leq g_{k}^{T}g_{k-1}\leq \|g_{k}{\|}^{2},$ Yousif [35] discussed the RMIL’s convergence result using the inexact line search Eqs (4) and (6). However, the Dai’s condition may not always hold for general functions.

Another way to ensure that a CG method produces descent search directions is to scale the first term of the search direction, i.e. −*g*_*k*_, with a positive parameter, usually referred to as *spectral parameter*. Such modifications are known as *spectral CG methods*. For instance, Raydan [36] introduced a spectral CG method with good convergence properties. Also, spectral FR CG methods were suggested in [37, 38]. The theoretical feature of these methods is that, regardless of which line search technique is employed, the direction *d*_*k*_ satisfies Eq (9). The method in [37] reduces to classical FR parameter provided Eq (3) is satisfied, while that of [38] did not explicitly mention the line search rule used. Unfortunately, their numerical results are not promising even with inexact line minimization. The following references are available for more information on hybrid and spectral CG methods [27, 38–47].

Inspired by the contributions discussed so far, a spectral RMIL version is proposed in this paper. Interestingly, it is worth nothing that one of the major advantages of the proposed spectral RMIL method is that it possesses the nice restart feature of PRP method as well as strong convergence property of FR method. This is evident from the theoretical analysis presented in Section 3. In addition, the proposed spectral RMIL method does not require the restriction imposed on the RMIL method by Dai [34]. Combining these features together, one can see that the spectral RMIL method works better than the earlier restricted versions of RMIL method.

The key contributions of this paper are enumerated as follows:

1. An efficient CG parameter is suggested from spectral search direction.
2. The search direction is always descent without any line search consideration at every iterations.
3. Several large-scale benchmark unconstrained optimization problems from the literature are used to demonstrate the efficiency of the suggested scheme.
4. The algorithm is shown to converge globally under Lipschitz continuity condition.
5. Finally, the arm robotic model and image restoration problems are solved using the new spectral method.

The reminder of the paper is structured as follows. The new spectral CG method and its algorithm is presented next. In Section 3, the global convergence result of the new scheme via the standard Wolfe line search is analysed. The experimentation and comparison are discussed in Section 4 as well as applications of the new algorithm to solve arm motion control and image restoration problems in section 5 and 6 respectively and finally brief conclusion is made in the last section.

## Algorithm and motivation

Consider the sequence {*x*_*k*_} generated by spectral CG method Eqs (2) and (7). Pre-multiplying Eq (7) by $g_{k}^{T}$ and using Eq (8) together with Cauchy-Schwartz inequality gives

$$\begin{array}{cc} g_{k}^{T}d_{k} & =-\theta_{k}\|g_{k}{\|}^{2}+\frac{g_{k}^{T}y_{k-1}}{\|d_{k-1}{\|}^{2}}g_{k}^{T}d_{k-1}\\ & \leq -\theta_{k}\|g_{k}{\|}^{2}+\frac{\|y_{k-1}\left\|\right\|d_{k-1}\|}{\|d_{k-1}{\|}^{2}}{\left\|g_{k}\right\|}^{2}\\ & =-(\theta_{k}-\frac{\|y_{k-1}\|}{\|d_{k-1}\|})\|g_{k}{\|}^{2}. \end{array}$$ (10)

For Eq (10) to satisfy Eq (9), implies that $\theta_{k}\geq \eta +\frac{\|y_{k-1}\|}{\|d_{k-1}\|}$, where *η* > 0. Without loss of generality, it holds when $\theta_{k}=\eta +\frac{\|y_{k-1}\|}{\|d_{k-1}\|},$ that is

$$\begin{array}{c} g_{k}^{T}d_{k}\leq -[\eta +\frac{\|y_{k-1}\|}{\|d_{k-1}\|}-\frac{\|y_{k-1}\|}{\|d_{k-1}\|}]\|g_{k}{\|}^{2}=-\eta \|g_{k}{\|}^{2}. \end{array}$$ (11)

Applying Cauchy-Schwartz inequality on Eq (11), we have

$$\begin{array}{c} \eta {\|g_{k}\|}^{2}\leq -g_{k}^{T}d_{k}\leq \left|g_{k}^{T}d_{k}\right|\leq \|g_{k}\|\|d_{k}\|, \end{array}$$

$$\begin{array}{c} \|d_{k}\|\geq \eta \|g_{k}\|\Rightarrow \;\;\;\frac{1}{\|d_{k}\|}\leq \frac{1}{\eta \|g_{k}\|}. \end{array}$$ (12)

Thus, base on the above selection of the parameter *θ*_*k*_, the search direction *d*_*k*_ is always descending independent of any line search rule, which means that the inequality Eq (9) is fulfilled. Therefore, motivated by this nice property, in this paper, we suggest the following spectral parameter

$$\begin{array}{c} \theta_{k}=\eta +\frac{\|y_{k-1}\|}{\|d_{k-1}\|}. \end{array}$$ (13)

**Remark 1**. *The choice of parameter θ*_*k*_ *in this form allows us to remove the computational burden and establish the global convergence of the proposed method without the condition*, $0\;\leq g_{k}^{T}g_{k-1}\leq \|g_{k}{\|}^{2}$ *imposed on the earlier versions of* $\beta_{k}^{RMIL}$.

In the following, the implementation procedure of the proposed method, *NSRMIL method*, is described.

**Algorithm 1: NSRMIL**

**Step 1**: Given an initial point $x_{0}\in R^{n}$. Compute *d*_0_ = −*g*_0_ and set *k* = 0.

**Step 2**: If ‖*g*_*k*_‖ ≤ *ϵ*, then stop. Otherwise,

**Step 3**: Compute *d*_*k*_ by Eq (7), where *β*_*k*_ and *θ*_*k*_ are determined by Eqs (8) and (13), respectively.

**Step 4**: Compute *α*_*k*_ > 0 satisfying Eqs (4) and (6).

**Step 5**: Set *x*_*k*+1_ = *x*_*k*_ + *α*_*k*_*d*_*k*_.

**Step 6**: Update the next iterate from step 2.

To analyse Algorithm 1 convergence characteristics, the following presumption is useful

**Assumption 2** *1. The function f*(*x*) *is bounded below on the level set* $\delta =\{x\in R^{n}:f\left(x_{0}\right)\geq f\left(x\right),\}$ *where x*_0_ *is the initial point such that*

$$\begin{array}{c} \left\|x\right\|\leq B,\;\;\forall x\in \delta ,\;B>0. \end{array}$$

*2. Denoting* Γ *as some neighborhood of δ, and function f is smooth with its gradient being Lipschitz continuous satisfying*

$$\begin{array}{c} \|g(x)-g(y)\|\leq L\|x-y\|,\;\;\forall x,y\in \Gamma ,\;\;L>0. \end{array}$$ (14)

Under these assumptions, it is easy to see that

$$\begin{array}{c} \|g_{k}\|\leq \gamma , \end{array}$$ (15)

for all *x* ∈ *δ*, *γ* > 0.

## Convergence analysis

We demonstrate the convergence of Algorithm 1 in this section. The Lemma that follows is taken from [25] and is an important part of the analysis.

**Lemma 3** *Suppose that* {*x*_*k*_} *and* {*d*_*k*_} *are sequences generated by Algorithm 1, where the search direction d*_*k*_ *is descent and α*_*k*_ *fulfils Wolfe condition, then*

$$\begin{array}{c} \sum_{k=0}^{\infty }\frac{{\left(g_{k}^{T}d_{k}\right)}^{2}}{{\left\|d_{k}\right\|}^{2}}<+\infty . \end{array}$$ (16)

**Theorem 4** *If Assumption 2 holds and the sequence of iterates* {*x*_*k*_} *is produced by Algorithm 1, then*

$$\begin{array}{c} \underset{k\to \infty }{\text{lim}\;\text{inf}}\|g_{k}\|=0. \end{array}$$ (17)

**Proof** If Eq (17) does not hold, then there exists some constant *m* > 0 so that

$$\begin{array}{c} \|g_{k}\|\geq m,\;\;\forall k\geq 1. \end{array}$$ (18)

**Claim**: There exists a constant *P* > 0, such that the search direction specified by Eq (7) is bounded.

$$\begin{array}{c} \|d_{k}\|\leq P,\;\;\forall k\geq 0. \end{array}$$ (19)

In order to establish the claim in Eq (19), which is crucial in showing the convergence of NSRMIL method, we need the following equations Eqs (8), (12), (13), (14), (15) and (18). Now taking the norm of the search direction given by Eq (7), we get

$$\begin{array}{cc} \|d_{k}\| & =\|-\theta_{k}g_{k}+\beta_{k}d_{k-1}\|\\ & \leq |\theta_{k}|\|g_{k}\|+|\beta_{k}|\|d_{k-1}\|\\ & =|\eta +\frac{\|y_{k-1}\|}{\|d_{k-1}\|}|\|g_{k}\|+\frac{|g_{k}^{T}y_{k-1}|}{{\|d_{k-1}\|}^{2}}\|d_{k-1}\|\\ & \leq \eta \|g_{k}\|+\frac{\|y_{k-1}\|}{\|d_{k-1}\|}\|g_{k}\|+\frac{\|y_{k-1}\|}{\|d_{k-1}\|}\|g_{k}\|\\ & =\eta \|g_{k}\|+2\frac{\|y_{k-1}\|}{\|d_{k-1}\|}\|g_{k}\|\\ & \leq \eta \gamma +2\frac{\|y_{k-1}\|}{\eta \|g_{k-1}\|}\gamma \\ & \leq \eta \gamma +2\frac{L\|x_{k}-x_{k-1}\|}{\eta \|g_{k-1}\|}\gamma \end{array}$$ (20)

$$\begin{array}{cc} & \leq \eta \gamma +2\frac{LB}{\eta m}\gamma . \end{array}$$ (21)

Setting $P=\eta \gamma +2\frac{LB}{\eta m}\gamma ,$ the result is established. The fourth and last line inequalities follow directly from Cauchy-Schwartz inequality and Assumption 2 respectively. Squaring both sides of Eq (11) gives

$$\begin{array}{c} {\left(g_{k}^{T}d_{k}\right)}^{2}\geq \eta^{2}{\|g_{k}\|}^{4}. \end{array}$$ (22)

Dividing through by ‖*d*_*k*_‖^2^ and summing gives

$$\begin{array}{c} \sum_{k=0}^{\infty }\frac{{\left(g_{k}^{T}d_{k}\right)}^{2}}{{\|d_{k}\|}^{2}}\geq \eta^{2}\sum_{k=0}^{\infty }\frac{{\|g_{k}\|}^{4}}{{\|d_{k}\|}^{2}}\geq \eta^{2}\sum_{k=0}^{\infty }\frac{\gamma^{4}}{P^{2}}=+\infty , \end{array}$$ (23)

which contradicts Eq (16). Thus, Eq (17) must hold.

## Numerical results

Here, we give the numerical output of Algorithm 1 using some set of test functions considered by Andrei [24] and Jamil [48]. The problems are presented in Table 1 and compared with the following methods:

- SRMIL+ [2]: Algorithm 1 with $\beta_{k}^{RMIL+}$ and $\theta_{k}=-\frac{g_{k-1}^{T}d_{k-1}}{\|d_{k-1}{\|}^{2}}+\beta_{k}^{RMIL+}\frac{g_{k}^{T}d_{k-1}}{\|g_{k}{\|}^{2}}$.
- SPRP [49]: Algorithm 1 with $\beta_{k}^{PRP}$ and $\theta_{k}=\frac{d_{k-1}^{T}y_{k-1}}{\|g_{k-1}{\|}^{2}}$.
- RMIL+ [35]: Algorithm 1 with $\beta_{k}^{RMIL+}$ and *θ*_*k*_ = 1.
- SCG [38]: Algorithm 1 with $\beta_{k}^{FR}$ and $\theta_{k}=-\frac{g_{k-1}^{T}d_{k-1}}{\|g_{k-1}{\|}^{2}}+\beta_{k}^{FR}\frac{g_{k}^{T}d_{k-1}}{\|g_{k}{\|}^{2}}$.

**Table 1:** List of the test functions.

NUMBER	TEST PROBLEM
1	EXT. PENALTY
2	EXT. MARATOS
3	DIAGONAL 5
4	TRECANNI
5	Q. PENALTY 1
6	Q. PENALTY 2
7	QUADRATIC F. 1
8	QUADRATIC F. 2
9	POWER
10	ZETTL
11	DIAGONAL 2
12	TEST
13	SUM OF SQUARES
14	SHALLOW
15	QUARTIC
16	MATYAS
17	DIAGONAL 1
18	HAGER
19	ZAPF
20	RAYDAN 1
21	RAYDAN 2
22	FLETCHER
23	DIAGONAL 3
24	EXT. DENSCHN B
25	DIAGONAL 6
26	DIAGONAL 4
27	DIAGONAL 7
28	DIAGONAL 8
29	DIAGONAL 9
30	DENSCHN A
31	DENSCHN C
32	EXT. B. DIAGONAL 1
33	HIMMELBLAU
34	DQDRTIC
35	QUARTICM
36	L.PERTURBED
37	TRID. WHITE AND HOST
38	ENGVAL 1
39	ENGVAL 8
40	DENSCHNF
41	ARWHEAD
42	SIX HUMP
43	PRICE 4
44	EXT. HIMMELBLAU
45	ROTATED ELLIPSE
46	EVF
47	EXT. HIEBERT
48	EXT. TRIDIAG 1
49	THUMP

To analyse the performance of these coefficients, we consider forty nine (49) set of problems with different dimensions, and the codes are written in MATLAB 9.12 (R2022a), which is run on a personal computer with configuration; Intel(R) Core i7–1195G7 PC, RAM of 16 GB and 2.90 GHz of CPU. In running the code, we set parameters *δ* = 10^−4^ and *σ* = 10^−3^ in the strong Wolfe line search conditions for all the algorithms. The algorithms are set to stop when ‖*g*_*k*_‖ ≤ 10^−6^ or 2000 iterations is reached. To determine the proper value for the modulating parameter, *η* in the NSRMIL scheme, we tested the numerical behavior of the parameter for some selected values {0.0001, 0.01, 0.5, 1, 10, 20, 50, 100}, where the best result is obtained when *η* = 0.01.

**Fig 1:**
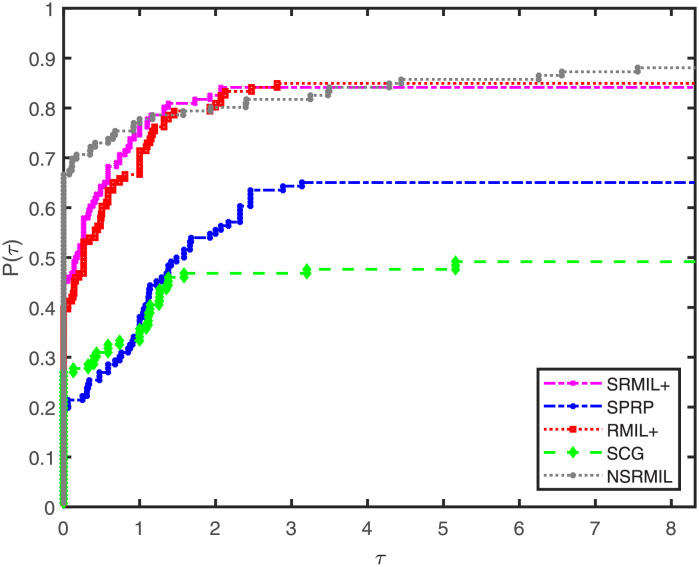
Number of iterations performance profiles of the methods.

**Fig 2:**
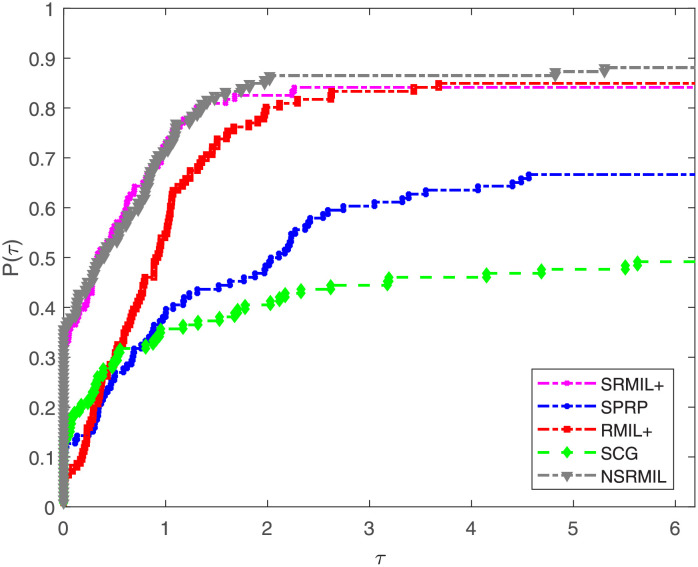
Time performance profiles of the methods.

**Fig 3:**
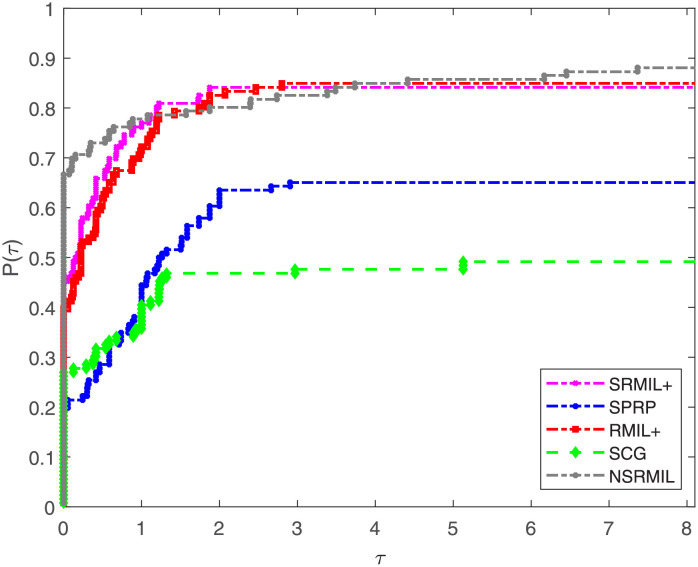
Number of function evaluation performance profiles of the methods.

Furthermore, numerical results are compared using performance profile introduced by Dolan and Moré [50]. The detailed description of the numerical outcomes of the experiments are tabulated and available in S1 Table, and the results are presented graphically in Figs 1–3, where the *P*(*τ*) in the graphs denote the fraction of the test functions a method solved, and a method having high performance ratio of *P*(*τ*) is regarded the best. In addition, this ratio measures the performance of the algorithms based on number of iterations (NI), number of function evaluation (FE) and amount of time required to solve the problems (CPU). Similarly, the right hand part of the curves indicate robustness of a method. The interpretation of Figs 1–3 show that NSRMIL is efficient and preferable to other four methods. In Figs 1–3 we can see that, the NSRMIL method solves 88% of the test problem and win, i.e be the best in about 68% of the problems, followed by SRMIL+, RMIL+, SPRP and SCG methods respectively.

## Application of NSRMIL on real-time 3DOF robotic model

In this section, we illustrate application of Algorithm 1 in solving the real-time motion control model of a three-joint planar robotic manipulator as investigated in [51]. The code and implementation of Algorithm 1 was performed using MATLAB R2022a 11^*th*^ Gen. Intel(R) Core i7–1195G7 and run on a PC with RAM 16 GB that has CPU of 2.90 GHz. Briefly, the position level of discrete-time kinematics model equation is written as

$$\begin{array}{c} h\left(\beta \right)=[\begin{array}{c} \eta_{1}\;\text{cos}\left(\beta_{1}\right)+\eta_{2}\;\text{cos}\left(\beta_{1}+\beta_{2}\right)+\eta_{3}\;\text{cos}\left(\beta_{1}+\beta_{2}+\beta_{3}\right)\\ \eta_{1}\;\text{sin}\left(\beta_{1}\right)+\eta_{2}\;\text{sin}\left(\beta_{1}+\beta_{2}\right)+\eta_{3}\;\text{sin}\left(\beta_{1}+\beta_{2}+\beta_{3}\right) \end{array}]. \end{array}$$ (24)

The relation implies that *h*(⋅) is the kinematics mapping, which relates the orientation and position of any part of the robot. The length of the segments is denoted by of *i*^*th*^*rod*, and *η*_*i*_ (for *i* = 1, 2, 3) and the vector $\beta \in R^{3}$ of *h*(*β*) is the joint angles that show the end effector position. Let $\delta_{k}\in R^{2}$ indicate the desired travel vector at time interval *t*_*k*_ ∈ [0, *t*_*f*_]. Therefore, in robotic model, we usually minimize the following nonlinear least square problem:

$$\begin{array}{c} \underset{\beta \in R^{3}}{\text{min}}\frac{1}{2}{\left\|h\left(\beta \right)-\delta_{k}\right\|}^{2}, \end{array}$$ (25)

where the vector *δ*_*k*_ represents the actual path. So, the end-effector is controlled to monitor Lissajous curve [52]:

$$\begin{array}{c} \delta_{k}=[\begin{array}{c} 1.5+0.4\;\text{sin}(\frac{\pi t_{k}}{5})\\ \frac{\sqrt{3}}{2}+0.4\;\text{sin}\left(\frac{\pi t_{k}}{5}+\frac{\pi }{3}\right) \end{array}]. \end{array}$$ (26)

**Fig 4:**
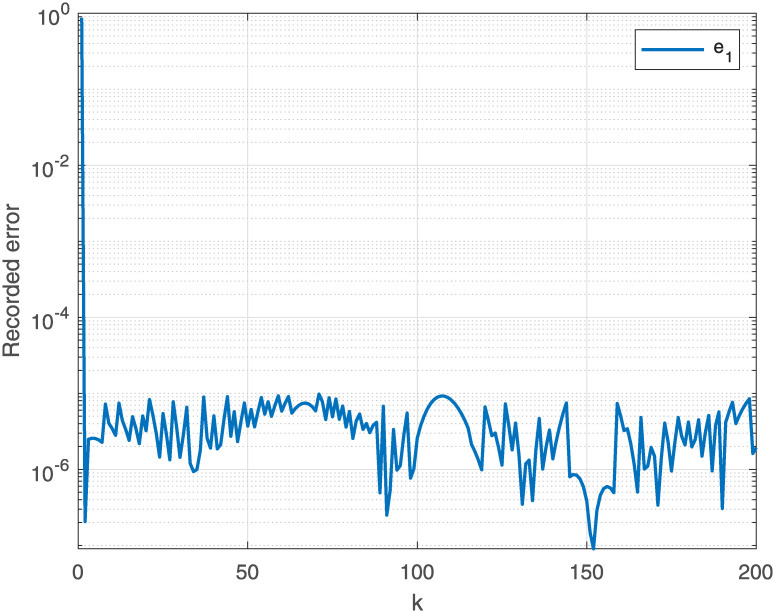
Error tracking by NSRMIL on x–axis.

**Fig 5:**
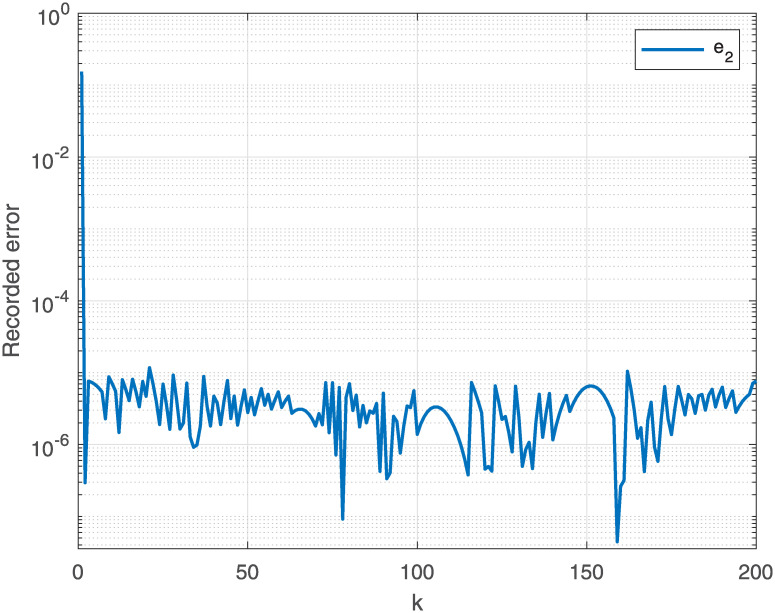
Error tracking by NSRMIL on y–axis.

**Fig 6:**
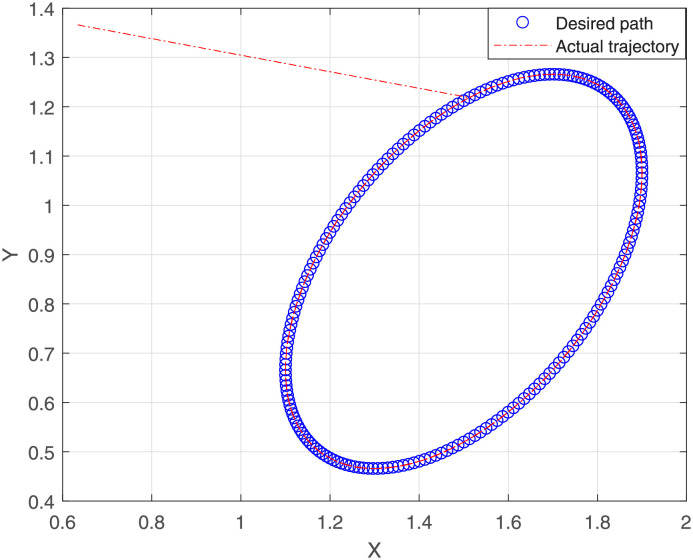
End effector of NSRMIL trajectory and desired path.

**Fig 7:**
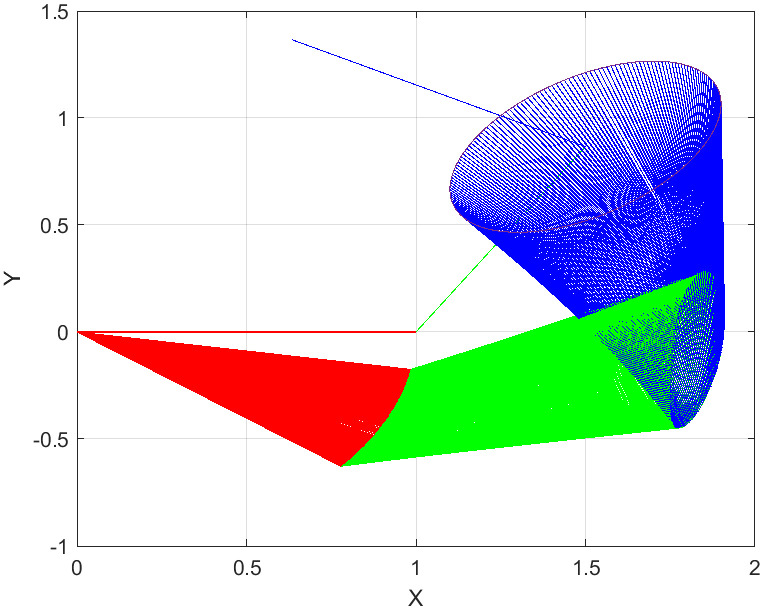
Robot trajectories synthesized by NSRMIL.

The joint is initialized at time instant *t* = 0, whereas the end effector *δ*_*k*_ at $\beta_{0}=\left[\beta_{1},\beta_{2},\beta_{3}\right]=\left[0,\frac{\pi }{3},\frac{\pi }{2}\right]$ with the task period [0, *t*_*final*_] that is equally divided into 200 units. The report of motion control experiment of Algorithm 1 are plotted in Figs 4–7, which show that, the NSRMIL method synthesized the robot trajectories and pass through the desired path as given in Figs 6 and 7 with residuals error of 10^−6^ as observed from Figs 4 and 5.

## Application of NSRMIL for image recovery

The conjugate gradient (CG) method is widely known for solving large-scale smooth and non-smooth convex optimization problems due to its efficiency and low memory requirement. A typical example of the smooth problem is the image restoration problem traced to the field of medical sciences, mathematical biology, and many more. This section tend to investigate the performance of Algorithm 1 in restoring images that have been corrupted by noise in the process of acquisition. One of the most common and frequently used noise restoration models is impulse noise (see; [53–55]). For the purpose of this study, we consider restoring the original 256 × 256 grey level images (*x*) of Canal and Building that have been corrupted by salt-and-pepper impulse noise. To achieve this, we need to define the following terms. From the above statement, we denote the true image with *M* × *N* pixels by (*x*) and its index set define as *W* = {1, 2, …, *M*} × {1, 2, …, *N*}.

At the first stage, suppose we denote the detected set of indices of the noise pixels as $K=\{\left(i,j\right)\in W|{\bar{\xi }}_{ij}\neq \xi_{ij},\;\xi_{ij}=s_{\text{min}}\;\text{or}\;s_{\text{max}}\}$. Then, if we want to restore the noise pixels in the second stage, we have to minimize the following function:

$$\begin{array}{c} \text{min}\;G(u), \end{array}$$

where,

$$\begin{array}{c} G\left(u\right)=\sum_{(i,j)\in K}\{\sum_{\left(m,n\right)\in V_{i,j}/K}\phi_{\alpha }\left(u_{i,j}-\xi_{m,n}\right)+\frac{1}{2}\sum_{\left(m,n\right)\in V_{i,j}\cap K}\phi_{\alpha }\left(u_{i,j}-u_{m,n}\right)\}. \end{array}$$

Here, *ϕ*_*α*_ denote the edge-preserving function defined as $\phi_{\alpha }\left(t\right)=\sqrt{t^{2}+\alpha }$ and *V*_*ij*_ = {(*i*, *j* − 1), (*i*, *j* + 1), (*i* − 1, *j*), (*i* + 1, *j*)} is the set of neighbours of the pixel at position (*i*, *j*). In the above function, $\bar{\xi }$ denote an adaptive median filter to the observed corrupted noisy image *ξ*.

In this part, the efficiency and relative accuracy of the proposed method is analysed based on peak signal-to-noise ratio (PSNR), relative error (RelErr), and CPU time (CPUT) using 30%, 50%, and 80% noise-degrees, respectively. The algorithms for this experiment are coded on MATLAB 2019a software installed on an Intel(R) Core(TM) i5–3210M PC with CPU@2.50 GHz, 4.00 GB RAM, and 64-bit.

**Fig 8:**
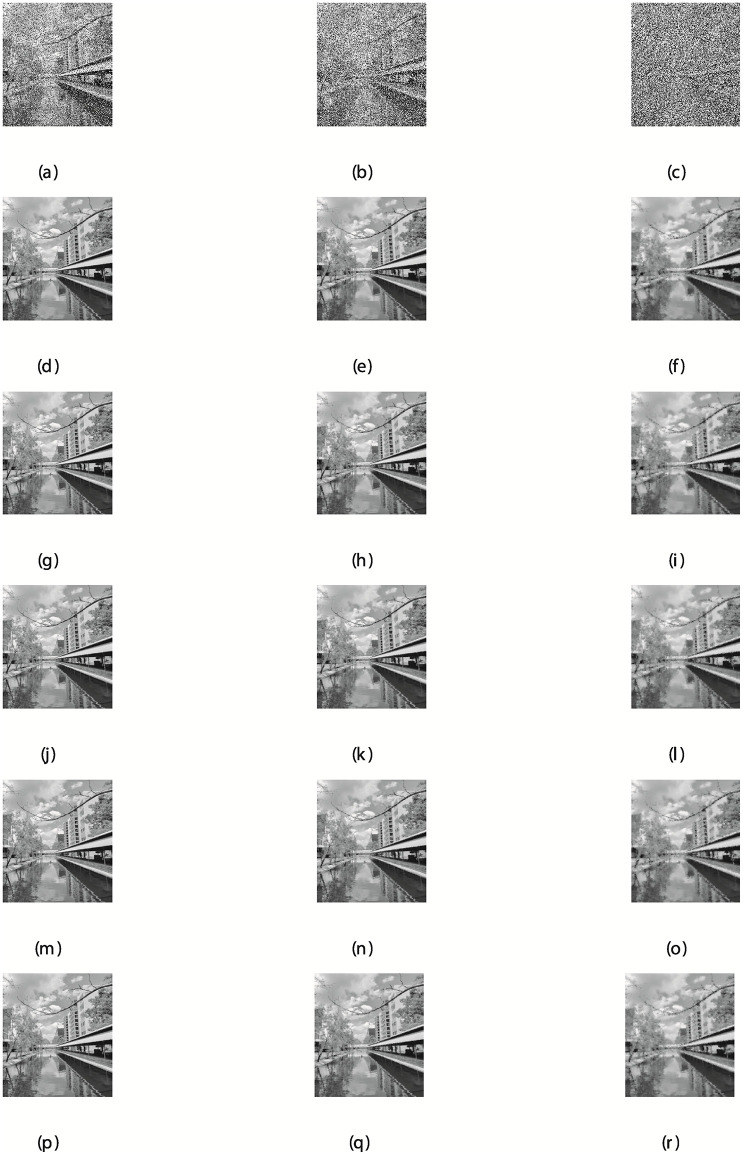
Canal image corrupted by 30, 50, and 80% salt-and-pepper noise: (a,b,c), the restored images using NSRMIL: (d,e,f), SPRP: (g,h,i), RMIL+: (j,k,l), SCG (m,n,o), SRMIL+ (p,q,r).

**Fig 9:**
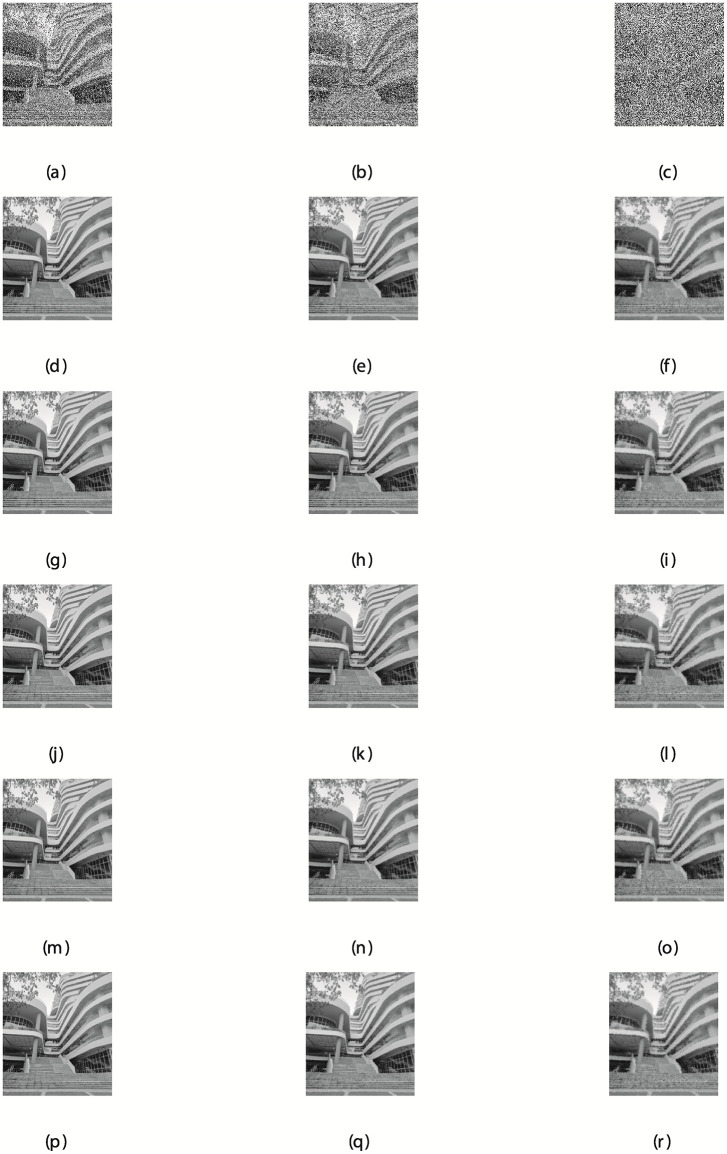
Building image corrupted by 30, 50, and 80% salt-and-pepper noise: (a,b,c), the restored images using NSRMIL+: (d,e,f), SPRP: (g,h,i), RMIL+: (j,k,l), SCG (m,n,o), SRMIL+ (p,q,r).

The detailed description of the results are discussed in Tables 2–4, and the graphical representation of the restored images are presented in Figs 8 and 9. The results presented in the tables and figures demonstrate the performance of all the methods considered for the study in removing the noise from the corrupted Canal and Building images. Considering the results as regards to the three metrics, including the PSNR, RelErr, and CPU time. It is obvious that the proposed method is very competitive because it produces the best performance, that is having the least CPUT for a greater number of the noise degrees as seen in Table 2. This also applies to other metrics analysed in Tables 3 and 4. A close observation of the overall results show that the proposed method significantly outperformed the existing algorithms based on CPUT, RelErr, and PSNR. The NSRMIL method is able to de-correlates the grey noise and improved the correlation in signals with better accuracy.

**Table 2:** Image restoration outputs for NSRIL, SRMIL+, SPRP, RMIL+, and SCG, based on CPUT.

METHODS		NSRMIL+	SPRP	RMIL+	SCG	SRMIL
**IMAGE**	**NOISE**	**CPUT**	**CPUT**	**CPUT**	**CPUT**	**CPUT**
	30%	40.9957	50.8202	53.6802	73.681	74.8004
**CANAL**	50%	83.0289	110.067	88.9899	117.339	106.171
	80%	216.4797	201.73	225.363	193.273	210.773
	30%	64.4833	51.3429	70.7243	48.3338	62.9352
**BUILDING**	50%	109.841	108.804	122.985	149.603	104.616
	80%	231.8347	266.189	297.651	259.953	234.514

**Table 3:** Image restoration outputs for NSRIL, SRMIL+, SPRP, RMIL+, and SCG, based on RelErr.

METHODS		NSRMIL+	SPRP	RMIL+	SCG	SRMIL
**IMAGE**	**NOISE**	**RelErr**	**RelErr**	**RelErr**	**RelErr**	**RelErr**
	30%	0.8219	0.8900	0.8008	0.8291	0.9404
**CANAL**	50%	1.2588	1.3592	1.2917	1.4658	1.3288
	80%	2.5543	2.5971	2.6327	2.7200	2.5891
	30%	1.4681	1.4052	1.4680	1.4885	1.4683
**BUILDING**	50%	2.6053	2.5924	2.4940	2.5574	2.6545
	80%	4.8644	5.0130	4.9616	5.3962	5.0617

**Table 4:** Image restoration outputs for NSRIL, SRMIL+, SPRP, RMIL+, and SCG, based on PSNR.

METHODS		NSRMIL+	SPRP	RMIL+	SCG	SRMIL
**IMAGE**	**NOISE**	**PSNR**	**PSNR**	**PSNR**	**PSNR**	**PSNR**
	30%	30.1102	31.1606	33.4670	31.3101	31.1762
**CANAL**	50%	27.8668	27.7682	27.9799	27.6228	27.6549
	80%	23.1146	23.2524	23.2273	22.9680	23.2032
	30%	29.8319	29.9003	31.1695	29.8805	29.7845
**BUILDING**	50%	26.4432	26.5135	26.5572	26.3321	26.4465
	80%	21.9663	22.4883	22.4338	22.0236	22.3539

## Conclusion

In this paper, we have presented a simple spectral CG by incorporating update parameter in [33]. The parameter is obtained by modifying the spectral CG direction, where the sufficient descent condition and the global convergence hold independent of any line search rule. The theoretical analysis is not based on assumptions of earlier $\beta_{k}^{RMIL}$ versions given in [2, 34, 35] respectively. The promising numerical result was obtained by relaxing the computational burden associated with the aforementioned versions. Similarly, the numerical result of the proposed algorithm is efficient, when the modulating parameter *η* = 0.01 compared to some known existing methods. The robustness of the NSRMIL is also demonstrated in solving image restoration problem and arm robotic planar problem. Future work include exploring the proposed method in two-step algorithms as presented in [56–58].

## Supporting information

S1 Table The numerical result of NSRMIL, SPRP, RMIL+ and SCG methods. The results presented in the table is used to demonstrate the performance of all the methods in plotting the graphs for the three metrics shown in Figs 1–3.
(XLSX)
